# Reactivation of Human Acetylcholinesterase and Butyrylcholinesterase Inhibited by Leptophos-Oxon with Different Oxime Reactivators *in Vitro*

**DOI:** 10.3390/ijms11082856

**Published:** 2010-08-03

**Authors:** Daniel Jun, Lucie Musilova, Miroslav Pohanka, Young-Sik Jung, Pavel Bostik, Kamil Kuca

**Affiliations:** 1 Faculty of Military Health Sciences, University of Defence, Hradec Kralove, Czech Republic; 2 Faculty of Environmental Sciences, Czech University of Life Sciences Prague, Praha, Czech Republic; 3 Faculty of Pharmacy in Hradec Kralove, Charles University in Prague, Hradec Kralove, Czech Republic; 4 Medicinal Science Division, Korea Research Institute of Chemical Technology, Yuseong, Taejon, Korea

**Keywords:** acetylcholinesterase, butyrylcholinesterase, nerve agent, pesticide, reactivator, oxime, scavenger, leptophos-oxon

## Abstract

We have evaluated *in vitro* the potency of 23 oximes to reactivate human erythrocyte acetylcholinesterase (AChE) and plasma butyrylcholinesterase (BChE) inhibited by racemic leptophos-oxon (*O*-[4-bromo-2,5-dichlorophenyl]-*O*-methyl phenyl-phosphonate), a toxic metabolite of the pesticide leptophos. Compounds were assayed in concentrations of 10 and 100 μM. In case of leptophos-oxon inhibited AChE, the best reactivation potency was achieved with methoxime, trimedoxime, obidoxime and oxime K027. The most potent reactivators of inhibited BChE were K033, obidoxime, K117, bis-3-PA, K075, K074 and K127. The reactivation efficacy of tested oximes was lower in case of leptophos-oxon inhibited BChE.

## 1. Introduction

Organophosphorus pesticides (e.g., chlorpyrifos, methamidophos) and nerve agents (e.g., sarin, tabun, VX) are highly toxic compounds for living organisms due to their ability to inhibit the enzymes acetylcholinesterase (AChE; EC 3.1.1.7) and butyrylcholinesterase (BChE; EC 3.1.1.8). Irreversible inhibition of AChE can result in cholinergic crisis and possible death of an intoxicated organism [1]. For the recovery of inhibited enzyme activity, oxime reactivators are usually used in combination with atropine [2,3]. The efficacy of reactivators is dependent on their chemical structure and also the type of inhibitor [4,5].

A relatively new approach for prophylaxis against such intoxication is the administration of a suitable scavenger capable of neutralizing organophosphates rapidly after their penetration into the bloodstream of the organism [6]. For these purposes, catalytic (e.g., phosphotriesterase, human paraoxonase) and stoichiometric (AChE, BChE) enzyme scavengers are now targets of intensive investigation. Furthermore, combinations of BChE with a suitable reactivator could be used as pseudocatalytic scavengers of organophoshorus pesticides or nerve agents [7–9].

In this study, we have evaluated the *in vitro* potency of 23 structurally different reactivators to reactivate human erythrocyte AChE and plasma BChE inhibited by racemic leptophos-oxon (*O*-[4-bromo-2,5-dichlorophenyl] *O*-methyl phenylphosphonate). The reactivation of BChE was tested in order to recommend suitable reactivators for preparation of pseudocatalytic scavengers. Leptophosoxon used in our experiments is a toxic metabolite of the pesticide leptophos, which is a weak AChE inhibitor itself. Racemic leptophos was previously employed to control insects in rice paddy fields and has been banned since 1978, after it was found to be highly neurotoxic to mammals [10,11]. It has been shown that enzyme inhibition potencies are different for the (+)- and (−)-isomers of leptophos. Thus, while no significant differences in IC_50_ for horse serum BChe were found between the (−)-leptophos and (±)-leptophos forms, the IC_50_ of the (+)-leptophos form was four times lower. Conversely, when leptophos-inhibited housefly head AChE was utilized, there was no significant difference in IC_50_ between the (+)-leptophos and (±)-leptophos forms, but the IC_50_ of the (−)-leptophos form was approximately two times higher [11]. We selected leptophos-oxon as a model organophosphorus inhibitor, as an appropriate representant of the organophosphorous pesticides that also belongs to the phosphonate group, which possess structural similarity with nerve agents.

**Figure 1:**
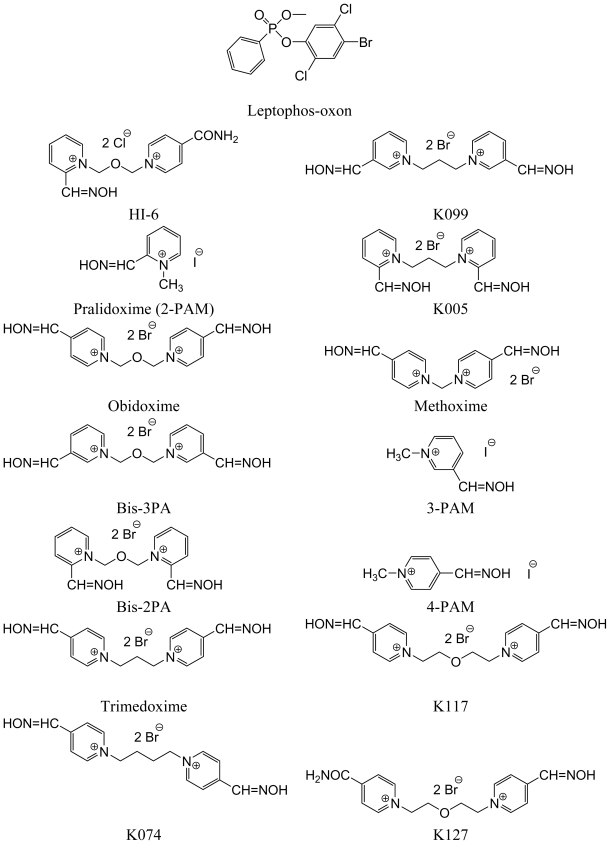

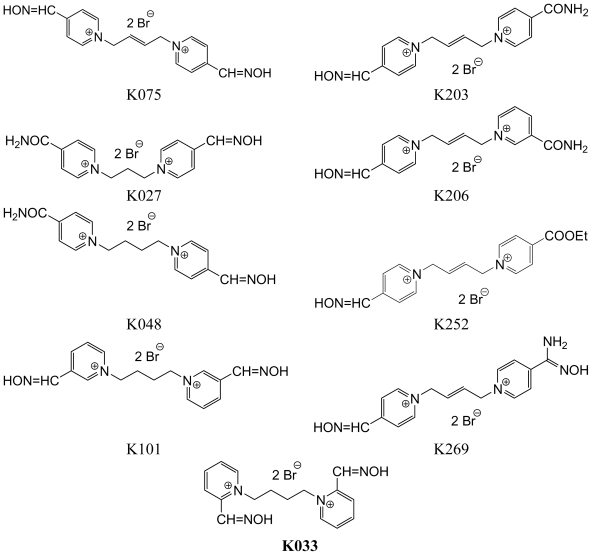
Structure of leptophos-oxon and tested oxime reactivators.

Oximes were assayed in concentrations of 10 and 100 μM, which are regularly attainable in plasma during antidotal treatment of pesticide intoxication [12]. Because this treatment requires administration of a fast and effective cholinesterase reactivator, we evaluated the reactivation ability of oximes over a 10-minute interval, which enabled us to compare this data with our previous results. In this study we tested both commercially available AChE reactivators and structurally different representatives of oximes (mono- and bis-quaternary with one or two oxime groups) previously synthesized in our lab with the aim to examine and elucidate the structure-activity relationships. The structures of leptophos-oxon and the tested oximes are shown below in Figure 1.

## 2. Experimental Section

Cholinesterase reactivators used in this study were synthesized in our lab or purchased from Leciva (Czech Republic), Merck (Germany) and Phoenix Chemicals Ltd. (United Kingdom). Purity of all the AChE reactivators utilized was tested using TLC (DC-Alufolien Cellulose F; mobile phase *n*-butanol-acetic acid-water = 5:1:2; detection by Dragendorff reagent) and NMR (Varian Gemini 300, Palo Alto, CA, USA). Racemic leptophos-oxon (*O*-[4-bromo-2,5-dichlorophenyl] *O*-methyl phenylphosphonate) of 95% purity was purchased from Dr. Ehrenstorfer (Augsburg, Germany). All other chemicals used in this study were of analytical purity and were purchased from Sigma-Aldrich (Czech Republic). The reactivation activity of synthesized reactivators was tested using our *in vitro* reactivation test [7–9].

Human erythrocyte hemolyzate was used as a source of AChE and human plasma as a source of BChE. Blood samples were collected from healthy volunteers into 3.8% sodium citrate (the ratio of blood/citrate was 1:10 w/w), centrifuged for 20 min at 2,856 g and the plasma removed as supernatant was stored at −80 °C (source of BChE). Erythrocytes were washed three times with the 0.1 M phosphate buffer (PB; pH 7.4), hemolyzed in the 0.01 M phosphate buffer (pH 7.4) in a ratio 1:10 (w/w) and frozen at −80 °C (source of AChE). The enzymes were inhibited to 5% of their original activity using concentration corresponding to IC_95_ (4.16 × 10^−7^ M for AChE and 7.06 × 10^−6^ M for BChE) in order to avoid the excess of leptophos-oxon. The IC_95_ value was determined in a concentration range 10^−3^–10^−9^ M using experimental conditions (buffer, time of inhibition, enzyme activity) identical to those used for the cholinesterase inhibition (described below). Identical reaction conditions were utilized for a determination of half- life (T_1/2_) of reaction between the enzyme and leptophos-oxon. The concentration of an inhibitor was 5 × 10^−6^ M and the AChE or BChE activities were measured in 5 min intervals. The time of enzyme inhibition used in the reactivation test was equivalent to 7 × T_1/2_ (120 min for AChE and 45 min for BChE). The inhibition of cholinesterase was started in plastic cuvette by the addition of inhibitor solution in isopropanol to the mixture of the 0.05 M phosphate buffer (pH 7.4) and hemolyzate (activity before inhibition was set to 10 U/L) or plasma (activity was set to 13.3 U/L). The concentration of isopropanol in the sample was 5% and had no significant influence on the activity of both cholinesterases. Control samples with an uninhibited enzyme were incubated for the appropriate time with isopropanol in final concentration 5%. Then the inhibited enzyme was incubated for 10 min with a solution of the reactivator in 0.05 M phosphate buffer (pH 7.4) at concentrations of 10 μM and 100 μM. The activity of AChE (BChE) was measured spectrophotometrically by the method modified according to Ellman with acetylthiocholine (butyrylthiocholine) as a substrate [9,13]. The final concentration of acetylthiocholine or butyrylthiocholine in the mixture was 1 mM. All results were corrected for hydrolysis of substrate by reactivators (oximolysis). Reactivation potency was calculated from the formula:

$$\%\text{R}=[1-(a_{0}-a_{\text{r}})/(a_{0}-a_{i})]\times 100$$

where%R is percent of reactivation, a_0_ is activity of intact enzyme, *a_i_* is activity of inhibited enzyme and a_r_ is activity of reactivated enzyme minus oximolysis.

Each measurement was repeated three times and was conducted at standard laboratory temperature (25 °C). All data are expressed as means with corresponding SD. Groups of data were compared using the one-way ANOVA test followed by the Tukey test. Calculations were performed using software GraphPad Prism version 4.00 for Windows (GraphPad Software, San Diego, CA, USA; www.graphpad.com). All experiments were carried out in compliance with the current applicable laws of Czech Republic.

## 3. Results

Measured values of reactivation potency of tested compounds are summarized in Table 1. Different superscript labels (e.g., ^a, b, c^) of reactivation percentage values in comparisons of series of compounds denote that measured potency is significantly lower than value measured for preceding oxime at *P* < 0.001 level.

The data showed that the reactivation potencies for AChE inhibited by leptophos-oxon at lower concentration of oximes (10 μM) decreased as follows: obidoxime (31.4%^a^), trimedoxime (26.4%^b^), K027 (16.4%^c^), K075 (14.2%^c^), K074 (12.6%^c^) and HI-6 (11.6%^c^). Other assayed oximes had reactivation potency below 10% at this concentration.

Different values were obtained, however, when a higher concentration of reactivators (100 μM) was used. At this concentration the best reactivation potency was achieved for methoxime (52.6%^a^), followed by trimedoxime (51.3%^a^), obidoxime (50.3%^a^), K027 (49.3%^a^), K075 (33.8%^b^), HI-6 (32.8%^b^), K203 (30.8%^b^), K074 (28.1%^b^) and K048 (26.1%^b^). Reactivation efficacy of other compounds was below 25%.

The results indicate that the most active reactivators were symmetrical bisquaternary compounds with two aldoxime or one aldoxime and one carbamoyl group at position 4 on each pyridinium ring. The optimal number of members in connecting chain was one or three, as the potency of oximes with four-member connecting chains was lower. Conversely, the monoquaternary compounds or bisquaternary compounds with oxime groups in position 2 on pyridinium rings (regardless of the length of the connecting chain) had relatively low potency. Out of the monoquaternary compounds tested, the most efficacious was pralidoxime (13.2% at 100 μM) with an aldoxime group in position 2, while other tested oximes were practically inactive (though they had the aldoxime group in position 4). From commercially available AChE reactivators, methoxime, trimedoxime, obidoxime and oxime HI- 6 were sufficiently effective.

Comparison of this data with the data from out previous study focused on the reactivation of AChE–inhibited by paraoxon shows, that obidoxime (96.9% of reactivation), trimedoxime (86.0%) [7] and compound K027 (86.0%) are the most effective reactivators evaluated in both studies [9].

The results obtained with the BChE showed that for this enzyme the best reactivation potency was achieved for obidoxime (6.5%^a^), followed by oximes K033 (4.2%^a^), bis-3-PA (2.7%^a^) and K117 (2.7%^a^) when lower concentration of the reactivators was used, while the other assayed oximes had reactivation potency below 2.5% at this concentration.

Similarly to the results obtained with AChE, different reactivation potencies were measured when higher a concentration of the reactivators was tested. Under these conditions, the reactivation ability of tested compounds decreased as follows: K033 (14.5%^a^), followed by obidoxime (14.3%^a^), K117 (13.9%^a^), bis-3-PA (13.1%^a^), K075 (12.4%^a^), K074 (11.5%^a^), K127 (11.0%^a^) and K269 (10.7%^a^). The reactivation efficacy of other compounds was below 10%.

Further analysis of this data together with our previous data on BChE inhibited by paraoxon showed that compounds K117 (16.4%), trimedoxime (12.2%), compound K269 (10.6%) and K075 (10.3%) were the most effective reactivators at the concentration of 100 μM [9]. The results also indicate that the most active reactivators of inhibited BChE were symmetrical bisquaternary compounds with two aldoxime groups and that the optimal number of members in connecting chain was three or four, while the monoquaternary compounds had low reactivation potency.

The likely reason for the significantly lower BChE reactivation ability at a higher concentration in comparison with AChE (*P* < 0.001) is that BChE lacks the reactivator aromatic binding pocket found in AChE, which itself is a part of the AChE peripheral anionic site. This difference finally renders the current AChE reactivators, when used with BChE, non-functional [14,15]. Overall reactivation ability of tested compounds at lower concentration was comparable for both cholinesterases with no significant difference found.

## 4. Conclusions

According to our results, human erythrocyte AChE inhibited by leptophos-oxon possesses very good ability for reactivation. Therefore, commercially available reactivators, commonly used in the therapy of OP intoxications, could provide sufficient efficacy in the treatment of poisonings. However, results obtained with BChE clearly showed, that none of the tested re-activators exhibited sufficient reactivation potency needed for the construction of a pseudocatalytic scavenger and therefore new compounds for this purpose need to be developed.

## Figures and Tables

**Table 1:** Potency of tested oximes to reactivate leptophos-oxon - inhibited human erythrocyte AChE and plasma BChE at concentrations 10 μM and 100 μM. (%, mean value of three independent determinations, time of inhibition by leptophos-oxon 120 min; time of reactivation by AChE reactivators—10 min; pH 7.4; temperature 25 °C). ^*^ *P* < 0.05, ^**^ *P* < 0.01, ^***^ *P* < 0.001, ns: not significant - compared with potency at 10 μM concentration.

	Reactivation (%)							
	AChE				BChE			

Concentration	10 μM		100 μM		10 μM		100 μM	

Reactivator	Mean	SD	Mean	SD	Mean	S.D.	Mean	SD
HI-6	11.6	0.4	32.8^**^	8.0	0	0	5.6 ^ns^	4.9
Methoxime	12.0	0.9	52.7^***^	0.5	1.9	1.8	6.4^**^	0.4
Obidoxime	31.5	0	50.3^***^	0.9	6.5	4.2	14.3^**^	0.6
Trimedoxime	26.4	2.7	51.3^***^	0.5	2.1	0.4	8.6^*^	2.4
Pralidoxime	4.1	1.3	13.3^ns^	0.9	0	0	2.3 ^ns^	1.8
3-PAM	0	0	0.3^**^	2.2	0	0	3.2 ^ns^	2.6
4-PAM	2.8	0.4	0.3^*^	0.4	0	0	4.7^***^	0.8
bis-2-PA	1.9	0.9	4.7^*^	1.3	2.3	0.7	5.3^*^	0.6
bis-3-PA	0	0	9.1 ^ns^	1.3	2.7	5.1	13.1^**^	1.5
K005	2.2	0.4	1.7^***^	1.4	0	0	0 ^ns^	0
K027	16.4	0.9	49.3 ^ns^	0.5	0	0	0 ^ns^	0
K033	6.3	0.9	4.1^***^	1.3	4.2	3.8	14.5^*^	5.6
K048	6.6	0.4	26.1^***^	0.4	0	0	3.9^***^	0.3
K074	12.6	0.9	28.2^***^	0.5	2.1	0.1	11.5^***^	1.5
K075	14.2	0.4	33.8 ^ns^	0	1.8	0	12.4^***^	1.9
K099	2.5	0.9	4.0 ^ns^	0.9	0	0	3.9^***^	0.8
K101	3.1	0.9	4.0 ^ns^	0	0.5	0.4	5.9^***^	0
K117	2.2	0.4	12.0^***^	1.8	2.7	0.1	13.9^***^	1.5
K127	0.6	2.7	12.6^***^	1.8	1.4	2.8	11.0^**^	0.8
K203	8.5	0.4	30.8^***^	0.9	0	0	8.4^***^	0.1
K206	7.6	0	20.1^**^	1.8	0	0	4.2 ^ns^	6.5
K252	1.6	0.4	15.4^***^	2.2	0	0	4.7^**^	0.6
K269	6.3	0.9	21.4^***^	0	0.5	0.4	10.7^***^	0.7
